# Potential of Oral Cavity Stem Cells for Bone Regeneration: A Scoping Review

**DOI:** 10.3390/cells12101392

**Published:** 2023-05-15

**Authors:** Josefa Alarcón-Apablaza, Ruth Prieto, Mariana Rojas, Ramón Fuentes

**Affiliations:** 1 Research Centre in Dental Sciences (CICO-UFRO), Dental School, Universidad de La Frontera, Temuco 4780000, Chile; 2 Doctoral Program in Morphological Sciences, Faculty of Medicine, Universidad de La Frontera, Temuco 4780000, Chile; 3 Department of Pediatrics and Pediatric Surgery, Faculty of Medicine, Universidad de La Frontera, Temuco 4780000, Chile; 4 Comparative Embryology Laboratory, Program of Anatomy and Developmental Biology, ICBM, Faculty of Medicine, Universidad de Chile, Santiago 8320000, Chile; 5 Department of Integral Adults Dentistry, Dental School, Universidad de La Frontera, Temuco 4780000, Chile

**Keywords:** stem cells, stomatognathic system, tissue engineering, bone regeneration

## Abstract

Bone loss is a common problem that ranges from small defects to large defects after trauma, surgery, or congenital malformations. The oral cavity is a rich source of mesenchymal stromal cells (MSCs). Researchers have documented their isolation and studied their osteogenic potential. Therefore, the objective of this review was to analyze and compare the potential of MSCs from the oral cavity for use in bone regeneration. Methods: A scoping review was carried out following the Preferred Reporting Items for Systematic Reviews and Meta-Analyses extension for Scoping Reviews (PRISMA-ScR) guidelines. The databases reviewed were PubMed, SCOPUS, Scientific Electronic Library Online (SciELO), and Web of Science. Studies using stem cells from the oral cavity to promote bone regeneration were included. Results: A total of 726 studies were found, of which 27 were selected. The MSCs used to repair bone defects were (I) dental pulp stem cells of permanent teeth, (II) stem cells derived from inflamed dental pulp, (III) stem cells from exfoliated deciduous teeth, (IV) periodontal ligament stem cells, (V) cultured autogenous periosteal cells, (VI) buccal fat pad-derived cells, and (VII) autologous bone-derived mesenchymal stem cells. Stem cells associate with scaffolds to facilitate insertion into the bone defect and to enhance bone regeneration. The biological risk and morbidity of the MSC-grafted site were minimal. Successful bone formation after MSC grafting has been shown for small defects with stem cells from the periodontal ligament and dental pulp as well as larger defects with stem cells from the periosteum, bone, and buccal fat pad. Conclusions: Stem cells of maxillofacial origin are a promising alternative to treat small and large craniofacial bone defects; however, an additional scaffold complement is required for stem cell delivery.

## 1. Introduction

Bone regeneration currently represents an important challenge in the field of regenerative medicine and craniofacial regeneration. Often in critically sized bone defects, the human body is unable to heal the bone on its own, which leads to nonunion and scar tissue formation [1]. Bone loss is caused by many diseases, trauma, and surgical procedures that lead to functionality problems, and its social impact is growing [2,3]. Autogenous bone grafting remains the gold standard for reconstructing bone defects; however, it is limited by the volume of bone that can be harvested, harvest site morbidity, local hematoma, and remodeling problems of the implanted bone [4,5]. Therefore, the limited supply of autogenous bone grafts and the risk of infection associated with allograft materials have prompted the search for an alternative approach to repair bone defects [1,4].

Bone regeneration is a complex process that requires the migration and proliferation of specific cells to the healing area in order to provide the biological substrate for new tissue growth [3,4]. For this approach, three essential components are typically required: (i) progenitor cells, to form tissues together with available host cells; (ii) stimulatory factors, to direct cellular processes; and (iii) a biomaterial template, to provide cells with a 3D cue to form tissue after implantation in vivo [4,6]. Stem cells are a promising alternative as they are a component of progenitor cells for bone formation that can be supplied exogenously [7]. Mesenchymal stromal cells (MSCs) are multipotent cells present in most adult connective tissues [8,9]. MSCs have the ability to promote better regeneration of soft tissues [10] and mineralized tissues [2]. They have been widely studied due to their ability to differentiate into multiple cell types [8]. Bone marrow (BM) is considered the main source of mesenchymal stem and progenitor cells (MSPCs) for experimental and clinical applications. However, due to the limited number of BM-MSPCs available for autogenous use, the implementation of alternative sources of MSPCs is particularly important [11,12].

Although there are several “loci” or “niches” within the adult human body made up of significant numbers of stem cells, these niches are often not easily accessible and have high residual anatomical site morbidity [13]. A number of studies have emerged which identified the presence of neural crest-derived stem cells (NCSCs) within different adult craniofacial tissues [14]. NCSCs may exist as a dormant multipotent stem cell population in the adult, as their pluripotent state becomes gradually more restricted after migration [14]. Due to their embryonic neural crest origin [11,15] and easy accessibility [16], intraoral tissues are a promising and rich source of stem cells for tissue engineering approaches with potential clinical applications [14], such as in regenerative dentistry [17]. In the oral cavity, stem cells can be isolated from various locations; among those that stand out are the dental pulp of deciduous and permanent teeth, dental follicle, apical papilla, periosteum, and periodontal ligament [1,7,18]. Dental stem cells are able to differentiate into osteoblasts, chondroblasts, and adipocytes [17,19]. Extensive research has been carried out to determine their differentiation mechanisms and efficacy in bone tissue regenerative medicine [1,18,20]. To date, different approaches have been used to induce bone repair in the injured area using stem cells from the oral cavity. However, despite the efforts made to describe the regenerative capacity of stem cells from the oral cavity, no exhaustive review has been found in the literature that details and compares the different sources of stem cells from the oral cavity and the bone regenerative results of each. This review aims to analyze and compare the potential of stem cells from different intraoral tissues for use in bone regeneration, focusing on the bone regenerative result achieved with stem cells from the oral cavity.

## 2. Materials and Methods

### 2.1. Systematic Literature Search 

A scoping review was performed on stem cells from the oral cavity used for bone regeneration. Our scoping review was performed according to the Preferred Reporting Items for Systematic reviews and Meta-Analyses extension for Scoping Reviews (PRISMA-ScR) guidelines [21].

An electronic search was carried out in four digital databases (PubMed, SCOPUS, Scientific Electronic Library Online (SciELO), and Web of Science). The search terms selected were: “stem cell *”, “progenitor cell *”, “autogenous periosteal cells”, “Mesenchymal Stem Cells”, “Mesenchymal Stromal Cells”, “Stem Cells” [Mesh], “Mesenchymal Stem Cells” [Mesh], “Multipotent Stem Cells” [Mesh], “Neural Crest Stem Cells”, “Bone Regeneration”, “Regenerative treatment”, “Regeneration, Guided Tissue”, “Bone”, “Formation *”, “Repair *”, “Densit *”””, “Tissue Regeneration”, “Guided Tissue Regeneration” [Mesh], “Bone and Bones” [Mesh], “Bone Density” [Mesh], “tooth,” “teeth”, “pulp”, “periodontal ligament”, “periosteum”, “Buccal Fat”, “apical papilla”, “deciduous tooth”, “dental follicle”, “oral cavity”, “dental papilla, “dental sac”, “Tooth” [Mesh], “Natal Teeth” [Mesh], “Tooth, Deciduous” [Mesh], “Dental Pulp” [Mesh], “Periodontal Ligament” [Mesh], “Periosteum” [Mesh], “Dental Papilla” [Mesh], and “Dental Sac” [Mesh]. The keywords were combined with Boolean terms OR and AND. The search was performed between May and December 2022. The bibliographies of potentially eligible clinical trials, case reports, case studies, and systematic reviews were also screened for any additional studies which were possibly fit for inclusion. The following search equation was used in PubMed: 

((((((((“stem cell *” [Title/Abstract]) OR (“Neural Crest Stem Cells”)) OR (“progenitor cell *” [Title/Abstract])) OR (“autogenous periosteal cells” [Title/Abstract])) OR (“Mesenchymal Stem Cells” [Title/Abstract])) OR (“mesenchymal stromal cells” [Title/Abstract])) OR (((“Stem Cells” [Mesh]) OR “Mesenchymal Stem Cells” [Mesh]) OR “Multipotent Stem Cells” [Mesh])) AND (((((((((“Bone Regeneration” [Title/Abstract]) OR (“Regenerative treatment” [Title/Abstract])) OR ((“Regeneration, Guided Tissue” [Title/Abstract]) AND (bone [Title/Abstract]))) OR ((Bone [Title/Abstract]) AND (“formation *” [Title/Abstract] OR “repair *” [Title/Abstract] OR “densit *” [Title/Abstract] OR “Regeneration *” [Title/Abstract]))) OR ((“Guided Tissue Regeneration” [Mesh]) AND (BONE))) OR ((“Tissue Regeneration”) AND (“Bone and Bones” [Mesh]))) OR ((“Regeneration” [Mesh]) AND (bone))) OR ((regeneration) AND (“Bone and Bones” [Mesh]))) OR ((“Bone Regeneration” [Mesh]) OR “Bone Density” [Mesh]))) AND (((((((((((((tooth [Title/Abstract]) OR (teeth)) OR (pulp)) OR (“periodontal ligament” [Title/Abstract])) OR (periosteum [Title/Abstract])) OR (“Buccal Fat” [Title/Abstract])) OR (“apical papilla” [Title/Abstract])) OR (“deciduous tooth” [Title/Abstract])) OR (“dental follicle” [Title/Abstract])) OR (“oral cavity”)) OR (“dental papilla” [Title/Abstract])) OR (“dental sac”)) OR ((((((((Tooth [Mesh]) OR “Natal Teeth” [Mesh]) OR “Tooth, Deciduous” [Mesh]) OR “Dental Pulp” [Mesh]) OR “Periodontal Ligament” [Mesh]) OR “Periosteum” [Mesh]) OR “Dental Papilla” [Mesh]) OR “Dental Sac” [Mesh])).

The same search equation was adapted for the other search engines. A summary of the factors considered in this review is presented in Table 1.

### 2.2. Eligible Criteria 

Observational (case reports and case series) and experimental (randomized and controlled clinical trials) studies were included where the general objective was to study stem cells from the oral cavity used for bone regeneration. The potentially eligible articles were screened based on the inclusion criteria: studies in English, Spanish, and Portuguese, full text with no publication date limit, and studies in which stem cells from the oral cavity were used for the treatment of bone defects. Animal and in vitro studies, studies using stem cells from a site other than the oral cavity, and articles not evaluating bone regeneration were excluded.

### 2.3. Article Selection and Data Extraction 

Two independent reviewers analyzed articles obtained in the systematic search process by reviewing the titles and abstracts. The articles that met the eligibility criteria were then analyzed in their full text to confirm their relevance. In cases of disagreement between the two reviewers, a third reviewer was invited to help resolve the differences of opinion. From the full-text articles that made up the final selection, relevant aspects of bone regeneration and stem cells from the oral cavity were compiled. The following information was collected and shown in Table 2: author, year of publication, study design, number of participants, sex and age of the subjects, source of origin of the stem cells, stem cells, bone defect treated, material/fact associated with stem cells, study groups, and the main result in bone regeneration. For Table 3, information was collected on the methodology and results of the studies, including the experimental procedure or isolation of stem cells, the post-surgical evaluation of the defect treated with stem cells, and the complications after treatment of the bone defect. The tables used in data extraction were designed by the authors of this review to obtain data relevant to the subject studied.

## 3. Results

### 3.1. Study Selection

**Figure 1:**
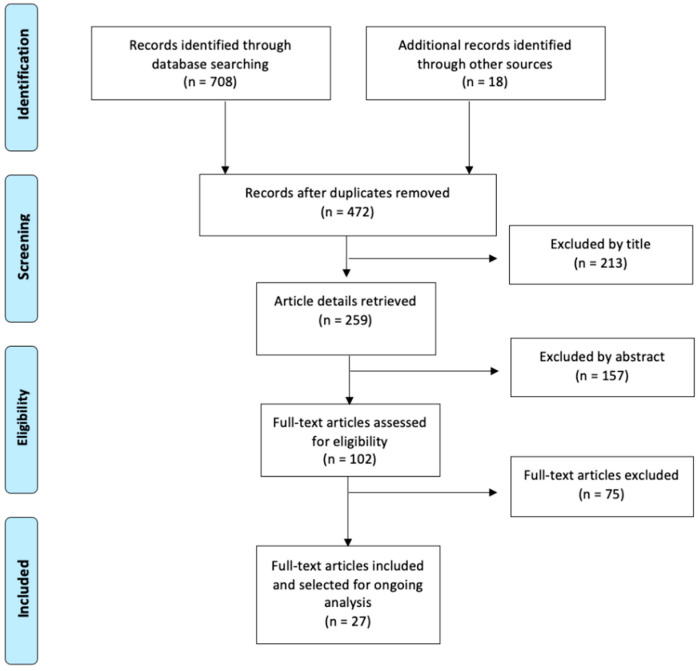
Flow chart for study selection.

The search and selection process for suitable articles is summarized in Figure 1. The total number of articles found in the databases was 708: 18 were identified from the manual search, and 254 articles were duplicates. After the initial reading by title, 213 were discarded, of which 84 were animal studies, 72 articles studied stem cells that did not come from the oral cavity, 47 were systematic reviews, and 10 were not related to the subject under study. After examination of the abstract, a further 157 studies were discarded, of which 89 studied stem cells that did not come from the oral cavity, 38 were studies that did not analyze bone regeneration, and 30 were not related to the subject of the review. After reading the full-text articles (102 articles), 75 were excluded, of which 37 studied stem cells that did not come from the oral cavity, 25 did not study bone regeneration, 13 were not related to the subject of study, and three were reviews of literature. A total of 27 articles corresponding to observational and experimental studies were finally included in this review.

### 3.2. Characteristics of the Selected Studies

This article analyzes the bone regenerative potential of stem cells from the oral cavity since they are a promising alternative to stem cell niches that are difficult to access. Data were extracted from human studies using Table 2 and Table 3, which detail the relevant information for studies examining the use of stem cells originating from the oral cavity for bone repair.

There have been extensive studies of bone regeneration using stem cells from the oral cavity in humans in the last two decades, beginning in 2003 [36]. The articles are mainly descriptive and observational: case reports [13,23,25,28,29,30,31,32,42,43], case series [24,27,41], or pilot studies [3,36,37,38,40]. However, in recent years, randomized clinical trials have been reported [2,18,22,26,33,34,35,39,44] which use stem cells from the oral cavity, obtaining promising results in bone repair.

Stem cells play an important role in bone repair. To this end, different niches in the oral cavity have been described as a source of stem cells; among them, the periosteum, from which periosteal cells are obtained [3,36,37]; deciduous teeth [29] and permanent teeth (mainly third molars and teeth with orthodontic extraction indication), to obtain periodontal ligament stem cells (PDLP) [30,31,32,33,34,35] and dental pulp stem cells [2,13,18,22,23,24,25,26,27,28]; buccal fat pads, for buccal fat pad-derived stem cells (BFPSCs) [38,39,40]; and cancellous bone, from which autologous bone-derived stem cells (H-MSVs) are obtained [41,42,43,44].

Tissue engineering allows the synthetic scaffold to be combined with stem cells to form hybrid constructs [45]. The analyzed studies have used different scaffold alternatives for seeding cells and to form a biocomplex to replace lost bone tissue. Collagen sponge is the most widely used biomaterial as a carrier for cell micrografts in bone regeneration [2,3,13,18,22,23,24,25,26,27,29,31,39]; however, there have been other promising alternatives with significant results, such as gelatin sponge [32], platelet-rich plasma [37], polymeric [36] and mineral-based biomaterials [28,30], an autogenous bone from the oral cavity [37,39] and the iliac crest [37,39], xenografts [33,35,41,42,43], and allografts [38]. A single study used isolated stem cells applied via a drip [40].

Stem cells obtained from the oral cavity have been used only to repair bone in oral and maxillofacial defects. The main condition studied has been the intraosseous periodontal defect [23,24,26,27,28,29,30,31,32,33,34,35], using stem cells from the dental pulp [23,24,26,27,28,29] and the periodontal ligament [31,32,33,34,35]. Other conditions in which bone repairs have been carried out with stem cells include the increase in the edentulous atrophic alveolus [3,36,37,38], elevation of the maxillary sinus [13,37,42] post-extraction alveolus or alveolar ridge [2,18,22,25,32], bone defect secondary to enucleation of cysts [40,41,44], and cleft lip and palate [39,43].

Cell culture was described by all the studies analyzed, in which four main modalities were described: processing by the Rigenera * system [3,13,18,25,27,31,38], cell culture in α-MEM [2,30,33,36,38,39] or DMEM [28,44], cell culture in osteogenic medium [40], and without cell culture, that is, where the stem cell-bearing tissue was immediately mixed with the scaffold [32,34].

The post-surgical evaluation of bone regeneration was evaluated through histological, radiographic, and clinical analysis. Information on clinical complications of the stem cell-grafted site was available in all studies. The complications evaluated were mainly signs of infection, such as pain [2,25,26,33,35,40], edema [2,3,25,31,40], inflammation [2,3,22,24,25,27,30,33,35,38,44], and functionality [2,3,22,25]. In addition, some studies evaluated healing of the area [2,3,33,36,37,39], paresthesia [40], foreign body reaction [38], and morbidity [2,18].

## 4. Discussion 

### 4.1. Stem Cells from the Oral Cavity in Bone Regeneration

**Figure 2:**
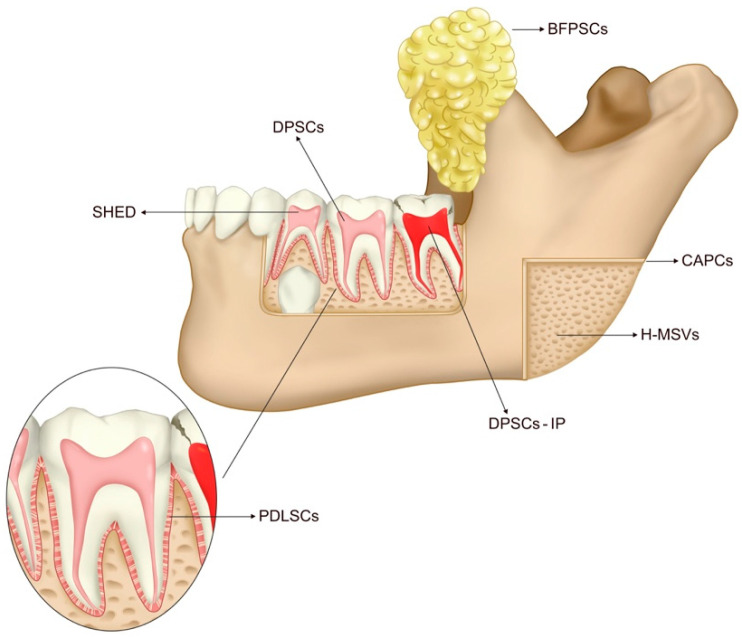
Oral cavity and its sources of stem cells for bone defect repair. Dental Pulp stem cells of permanent teeth (DPSCs) are derived from the healthy dental pulp of permanent teeth. Stem Cells Derived from Inflammatory Dental Pulp (DPSCs-IP) derived from inflammatory dental pulp. Stem cells from exfoliated deciduous teeth (SHED) are derived from the healthy dental pulp of deciduous teeth. Periodontal Ligament Stem Cells (PDLSCs) are derived from the periodontal ligament. Cultured Autogenous Periosteal Cells (CAPCs) derived from the periosteum. Buccal Fat Pad Derived Cells (BFPSCs) derived from the buccal fat pad. Autologous Bone-derived mesenchymal stem cells (H-MSVs) derived from bone.

MSCs can be isolated from various cellular niches, and some of the most accessible ones are located in the oro-maxillo-facial (OMF) area. In the oral cavity, stem cells of dental origin, such as dental pulp stem cells and periodontal ligament stem cells, can be found, which are exclusive to this area and which exhibit features of NCSCs [14]. Additionally, stem cells that are not exclusive to the oral cavity can also be found in other structures of the body, such as cultured autogenous periosteal cells, fat-derived cells, and autologous bone-derived mesenchymal stem cells. Figure 2 illustrates the origin of oral cavity stem cells used to repair bone defects.

The use of oral cavity stem cell therapy for bone regeneration has been extensively studied through in vivo experiments. Animal studies have shown the efficacy of MSCs derived from the oral cavity, such as from dental pulp [46], periodontal ligament [47], and periosteal cells [48], in bone regeneration. In the last two decades, their effectiveness in humans has been demonstrated. Table 4 summarizes the types of stem cells from the oral cavity and the associated scaffolds used to insert them into the bone defect that have been employed in human studies for the regeneration of bone tissue.

### 4.2. Dental-Origin Stem Cells

#### 4.2.1. Dental Pulp Stem Cells 

Dental pulp stem cells (DPSC) were derived from the dental pulp of permanent and deciduous teeth. The differentiation capacity of dental pulp tissue has been extensively studied since they were first identified by Gronthos et al. in 2000 [49]. The first report on DPSCs revealed that their properties are comparable to those of stem cells from the bone marrow (BMSC) in vitro and in vivo [49,50]. DPSCs have been used to regenerate structures within the oral cavity and elsewhere. They can be helpful both for the regeneration of soft tissue components and for the regeneration of mineralized structures [51]. Human pulp stem cells include dental pulp stem cells isolated from dental pulp tissues of extracted permanent teeth, stem cells derived from inflamed dental pulp, and stem cells from human exfoliated deciduous teeth.

##### Dental Pulp Stem Cells of Permanent Teeth

Dental pulp stem cells (DPSCs) cells of permanent teeth are used in the repair and regeneration of bone, periodontal intrabony defects, and dental defects [19]. It is an easily accessible source of MSCs derived from the dental pulp of caries-free third molars [2,13,18,22,23,24,25,26] (teeth in need of extraction due to impaction or poor positioning [27,28]) or teeth supernumeraries [28]. The dental pulp is easily collected using sterile Gracey curettes after root–crown separation to open the pulp chamber and expose pulp tissue [13,23].

Papaccio et al. [52] have conducted several studies on dental pulp stem cells (DPSCs) and have found that these are mainly multipotent cells that can be safely cryopreserved. DPSCs widely proliferate, with a doubling time of 24 h [53], and can have a long lifespan, up to 2 years after cryopreservation [52]. Dental pulp stem cells have been shown to express the MSC markers STRO-1, CD90, CD29, CD44, CD166, CD105, CD106, CD146, CD13, and are also negative for CD14 and CD34 [45].

The classical approach for bone regeneration requires a synthetic or natural scaffold for the implantation of stem cells in the bone defect [54]. All the studies analyzed used DPSCs seeded onto a collagen-based sponge scaffold [2,13,18,22,23,24,25,26,27]. This allows the formation of a biocomplex constituted by the collagen sponge as a carrier of cell micrografts that has no radiopacity at all [31]. Various studies have amply demonstrated that if DPSCs have seeded on a collagen I scaffold, the resulting biocomplex will allow the formation of well-differentiated bone of critical sizes [3,13,22,25,26].

Different bone repairs of the oral cavity with DPSCs have been reported, such as infra bony periodontal defects [23,24,26,27], post-extraction sockets [2,18,22,25], and maxillary sinus lifts [13]. For deep periodontal intrabony defects [23,24,27], the application of DPSCs significantly improved the clinical parameters of periodontal regeneration after one year of treatment compared with a defect treated without DPSCs [26]. On the other hand, DPSCs have been used for larger bone defects such as impacted lower third molar (ITM) post-extraction sockets. Barbier et al. [18] found no significant differences in the clinical, radiological, and surgical characteristics of the ITM between the groups treated with and without cells. However, it was shown that DPSCs allowed the formation of well-differentiated bone in post-extraction sockets with the formation of the Haversian system containing a critical amount of bone tissue [25]. Studies by d’Aquino et al. [2] and Giuliani et al. [22] evaluated alveolar repair secondary to third molar impaction. Using clinical and radiographic analysis, d’Aquino et al. [19] determined that at three months, there was greater clinical insertion compared with the group treated without cells. Giuliani et al. [22] evaluated the same subjects after 3 years, at which time greater bone hardness and less exposure of molar roots were observed compared to cell-free sites. Therefore, the dental pulp can be considered an interesting and potentially important source of autologous stem cells for therapeutic use in craniofacial bone regeneration.

##### Stem Cells Derived from Inflamed Dental Pulp

The discovery of DPSCs has provided new perspectives for bone tissue repair. However, a limitation for clinical application is the availability of DPSCs since these come from healthy tissue. Recently, some studies found that a certain proportion of ectomesenchymal stem cells were contained within inflamed tissues of the dental pulp, and that these had the potential for tissue regeneration [55,56,57]. Inflammation is a complex process that varies widely from one individual to another. Depending on the intensity of the inflammation, some stimuli can activate some stem cell properties, thus inducing their proliferation and differentiation. Hypoxia has been shown to increase DPSC proliferation [55,56,57,58,59] and the angiogenic potential of dental pulp cells [60]. Pereira et al., 2012 [61] compared cells of normal and inflamed human dental pulp and found that the morphology, proliferation rate, and differentiation potential of inflamed DPSCs were similar to those observed for normal DPSCs, thus demonstrating that the inflammatory process did not affect the stem cell properties that were assessed. However, Li et al. [28] later determined that the proliferative and osteogenic differentiation capacity of DPSC-IPs was slightly decreased, while the adipogenic and chondrogenic differentiation capacity did not show any significant differences compared with normal DPSCs (DPSC-NP). Therefore, to a certain extent, DPSC-IPs preserved the properties of DPSCs, including the expression of certain surface markers of mesenchymal stromal cells. DPSC-IPs showed highly positive expression levels of CD44 and CD90, while the levels of CD34 and CD45 were negative, in line with characteristics of mesenchymal stromal cells [28]. Previous studies have shown that although they lose some of the properties of stem cells, DPSC-IPs retain the potential for tissue regeneration [28,55,56]. These results suggested that although osteogenic capacity was impaired to some extent, DPSC-IPs could still be successfully cultured and amplified for the replacement of DPSC-NPs in clinical practice. Li et al. [28] provided evidence that DPSC-IP/β-TCP compounds may have a certain repair effect on periodontal hard tissue defects caused by periodontitis and may be a new source of oral tissue regeneration for potential future clinical applications.

##### Stem Cells from Exfoliated Deciduous Teeth 

Stem cells from exfoliated deciduous teeth (SHEDS) are DPSCs derived from human exfoliated deciduous teeth. In 2003, Miura et al. [7] performed the first isolation of a population of MSCs from the pulp tissue of the crown of exfoliated deciduous teeth. It was identified that SHEDs are a population of highly proliferative postnatal stem cells [7,62] capable of differentiating into a variety of cell types with neurogenic [63,64], adipogenic, odontogenic, and osteogenic potential [7,64,65,66,67,68]. The degree of bone regeneration with SHEDS relative to the bone defect is almost equivalent to that with BMSCs [69]. Kunimatsu et al. [67] determined by in vitro experimentation that SHEDS exhibit greater proliferative activity, odontogenic and osteogenic differentiation potential, and osteoinductive capacity compared with DPSCs from permanent teeth.

Kim et al. [70] and Vakhurushey et al. [71] found that in vitro osteogenic differentiation of SHEDS enhances hard tissue formation when transplanted subcutaneously [70,71]. Similarly, SHEDS produce mineralized structures in vivo. SHEDS effectively repaired orofacial defects of critical size in animal models such as mice [66] rats, [65,72] minipig [68], and dogs [62] without any immune reaction [62,65]. Behnia [62] and Ma et al. [73] showed in their in vitro and in vivo experiments that cryopreservation of SHEDS for more than two years did not affect their multipotent properties and that SHEDS could be successfully used as a therapeutic approach. Thus, from a practical perspective, stem cells from deciduous teeth were an easily accessible, widely proliferating source of autologous stem cells capable of engrafting and regenerating bone to repair bone defects of critical size, indicating that SHEDs constitute a promising model for possible therapeutic applications [7,66,72,74]. Due to the few bone lesions in children, SHEDs have not been used in infants; however, a study has described their osteoregenerative capacity as an allograft [29]. Despite the significant osteoinductive results observed with SHEDS in vitro and animal models, further experimental studies are required to demonstrate their regenerative capacity in humans, as only one case report has been published.

#### 4.2.2. Periodontal Ligament Stem Cells

The potential of periodontal ligament (PDL) stem cells was first described by Seo et al. [75]. These were isolated from extracted human third molars and transplanted into immunocompromised mice and rats to assess their regenerative potential [75]. Since then, numerous in vitro and in vivo studies have been performed to further evaluate the regenerative capacity of PDL stem cells [76,77,78]. This has been followed by experimental applications to study its clinical efficacy in humans. The human PDL contains a group of stem cells (PDLSCs) that express the surface markers of MSCs, present self-renewal capacity, and have multipotent capacity. PDLSCs are the most studied source and are considered the most suitable for periodontal intrabony defects [31]. These cells are easily accessible from the adherend of the extracted tooth roots [34] and are capable of secreting the mineralized structure [14]. The third molars have mainly been used to obtain periodontal tissue [30,32,33,34,35].

Ex vivo cultured periodontal ligament stem cells (PDLSCs) isolated from soft tissues adherent to extracted teeth have shown the ability to regenerate periodontal intrabony defects in animal models [79], a finding that has also been replicated in human studies [30,31,32,33,34,35]. Pilot studies, randomized controlled trials (RCTs) [33], and case reports [31] have demonstrated the potential of PDLSCs to be a powerful tool for periodontal intrabony therapy.

Intraosseous pockets, which result from bacterial infection and lead to bone resorption, are bone defects in the periodontal complex. Although adequate therapy can resolve the infection, it is not always possible to restore the injured tissue [31]. However, PDL tissue-derived cells have been shown to have the ability to regenerate alveolar bone tissue [30,31,32,33,34,35]. This regenerative capacity is attributed to a small number of progenitor cells within the PDL that retain their potential for proliferation and differentiation. To promote this regenerative potential, these stem cells must be combined with scaffolds made from various biomaterials, such as xenogenic bone substitute (XBS) [33,35], gelatin sponge [32,34], CALCITITE 4060-2 bone graft material [30], or collagen sponge scaffold [31].

Although all patients in the studies analyzed showed clinical benefits after PDLSC transplantation, no statistically significant differences in clinical parameters were detected between the cell group and the control group [33,34,35]. However, the radiographic analysis revealed a significant difference in bone defect density [34] and mineralization rate [31] in the cell-treated groups. These improvements in defect area and density are promising results of PDLSC application for the treatment of periodontal intrabony defects [33,35].

### 4.3. Non-Dental Origin Stem Cells from the Orofacial Region

#### 4.3.1. Cultured Autogenous Periosteal Cells 

In 1742, Duhamel was the first investigator to study the osteogenic potential of the periosteum. A century later, Ollier discovered that the transplanted periosteum could induce new bone formation. Based on the studies mentioned above and the advances in cell culture, H.B. Fell, in 1932, was the first to report the culture of the periosteum and its cells. Fell used an in vitro experiment to determine the ability of this tissue to form mineralized tissue. In the 1990s, the research group of A.L. Caplan pioneered the in vivo investigation of the osteogenic potential of periosteal cells in the field of bone engineering [80].

The periosteum is a highly vascular connective tissue that covers bone surfaces. It is composed of an external fibrous layer containing elastic fibers and micro vessels and an inner cambium layer where periosteum-derived progenitor cells (PDPCs), major players in bone development and fracture healing, reside [81]. Periosteal cell micrografts have been shown to maintain high cell viability and high positivity for stem cell markers such as CD73, CD90, and CD105 [82]. Three studies have looked at periosteal-derived autologous cells for bone regeneration [3,36,37]. Cultured autogenous periosteal cells (CAPCs) have been used for alveolar ridge augmentation [3,37], edentulous atrophic posterior maxillary alveolus [36], and maxillary sinus lift repair [37] in combination with biocompatible materials in specific collagen membranes soaked in cell suspensions to build a biocomplex that can be grafted directly onto the site [3].

A study by d’Aquino et al. [3] revealed significantly lower overall resorption of the alveolar ridges after extraction of a multi-rooted tooth in the group treated with periosteal cells and collagen compared with that treated solely with collagen, achieving 36% less horizontal and vertical resorption of than the group treated with collagen. Furthermore, it has been shown histologically [3] and radiographically [37] that the ossification process was much faster in the group treated with these cells at 45 days compared with the control group without cells. On the other hand, Nagata et al. [37] mixed CAPCs with particulate autogenous bone and platelet-rich plasma and achieved satisfactory results, even in cases of advanced atrophy, revealing prominent recruitment of osteoblasts and osteoclasts accompanied by angiogenesis around the regenerated bone. Therefore, the use of stem cells derived from the periosteum offers bone formation and remodeling with successful results, allowing the reduction of autogenous bone content if used as a complement to the cells [37]. This makes the procedure less invasive, and it is even possible to completely dispense with the use of autogenous bone and use collagen matrices instead [3,37]. In addition, the periosteum is freely accessible through the superficial layer of the oral cavity throughout its lifespan, and this is another important advantage of the use of the periosteum [37].

#### 4.3.2. Buccal Fat Pad-Derived Cells

Adipose stem cells (ASCs) were first discovered in 2001 by Zuk et al. [83] and are now widely used in tissue engineering. Their advantage over other sources is that they are generally obtained from disposable liposuction tissues, and some studies have found their properties comparable to those of bone marrow-derived stem cells (BMMSCs) [84]. The buccal fat pad (BFP) is an ideal tool in the hands of an oral and maxillofacial surgeon for tissue engineering for bone tissue repair [40]. Promising results have been obtained in bone defects produced by the enucleation of cysts and tumors [40], cleft lip and palate [39], and atrophic alveolar ridges [38]. BFP was harvested from healthy subjects through a buccal incision distal to the maxillary second molar [38]. To isolate BFPSCs, 3 to 10 mL is excised under aseptic conditions [38,39]. BFPSCs have the capacity for osteogenic differentiation in vitro and have shown good adhesion to scaffolds [38]. In humans, BFPSCs have been applied in different ways to bone defects. Meshram et al. [40] collected the BFPSCs and applied them via a drip to fill the bone defect left by the enucleation procedure in a dry surgical field [40]. Khojasteh et al. [38] and Khojasteh et al. [39] used an allograft and a collagen membrane with autograft, respectively, to fill the bone defect.

Three studies demonstrated the feasibility of reconstructing bone defects with BFPSCs [38,39,40]. Meshram et al. [40] observed an increase in bone density between the preoperative and postoperative stages, going from thick irregular trabecular bone in the first month to dense compact bone at six months [40]. On the other hand, two studies by Khojasteh et al. [38,39], achieved a significantly higher percentage of newly formed bone in the BFPSC-treated group compared with the control. Therefore, the application of MSCs derived from buccal fat pads together with different scaffolds is promising for bone repair [38]. However, age is an important factor to consider in the effectiveness of this treatment. The total number of cells in the oldest patient was lower and took the longest time to culture compared with samples from younger patients [40,44]. This suggests that with increasing age, the proliferative capacity of stem cells deteriorates.

#### 4.3.3. Autologous Bone-Derived Mesenchymal Stem Cells 

MSCs can be isolated by minimally invasive means from craniofacial bone, including alveolar bone [12,83,84]. Alveolar bone stem cells have osteogenic potential [12] and immunomodulatory properties comparable to those of bone marrow-derived stem cells commonly used in bone regeneration (BMMSCs) [12,85].

Tissue engineering that combines a scaffold with mesenchymal stromal cells derived from cancellous bone has shown excellent results for the repair of bone defects in animal models [85,86,87]. For this reason, Redondo et al. [44] and Pradel et al. [41] presented clinical trials using autologous bone-derived mesenchymal stem cells (H-MSVs). Redondo et al. 2017 [44] obtained H-MSVs from the intraoral bone using a 2 mm trephine cultured on a serum cross-linked scaffold (BioMax) for the treatment of maxillary cysts. Biomax favors cell nesting and growth and is very well tolerated by the host [88]. Two to four disks were used for each cystic bone defect [41,44]. A significant increase in computed tomography (CT) density inside the cyst after treatment could be observed. By contrast, the density of the control area did not present changes.

Pradel et al. has investigated different bone defects using autologous bone-derived mesenchymal stem cells [41,42,43]. They transplanted stem cells from jaws into an enucleation of cysts [41]. By radiographic analysis, the group treated with stem cells showed considerably greater ossification in cystic cavities grafted with autogenous osteoblasts in collagen-based scaffolds [41]. In 2008, Pradel et al. investigated sinus lift using stem cells obtained from the maxilla seeded in the demineralized bone matrix (DBBM) and solvent-dehydrated mineralized bovine bone (SDBB), achieving better results in SDBB [42]. Subsequently, in 2012, Pradel and Lauer [43], using the same cell culture, achieved greater ossification of the bone defect in the test group compared with the control using spongeous iliac bone. Therefore, cell therapy with H-MSVs associated with a scaffold could be considered as an alternative for bone defects.

### 4.4. Mesenchymal Staminal Cell Biomarkers

Stem cells obtained from the oral cavity are characterized by the negative expression of hemopoietic antigens such as CD14, CD19, CD24, CD34, CD45, and HLA-DR, and positive expression of mesenchymal stromal cell markers such as CD10, CD13, CD29, CD44, CD73, CD90, and CD105 (Table 5) [89,90,91,92,93]. The biomarkers expressed in stromal cells can vary depending on their origin and state of differentiation. Even within the same source of stem cells, the expression of biomarkers may present variations, as observed in the studies analyzed. The osteogenic biomarkers of oral cavity stem cells indicate their ability to differentiate into bone cells and, therefore, their potential for bone regeneration (Table 5).

MSCs are capable of differentiating into various cell types, including bone cells, and have the potential to regenerate damaged tissues and bone structures. The mechanisms of action of stem cells for osteogenesis mainly involve cell differentiation, as they have the ability to differentiate into osteoblasts and produce bone matrix. This process is favored by growth factors such as TGF-β (Transforming Growth Factor-β), BMPs (Bone Morphogenetic Proteins), and PDGF (Platelet-Derived Growth Factor), which are found in the local environment of the lesion and the expressed biomarkers [94].

The reviewed studies that used CAPCs and H-MSVs for bone regeneration did not assess bone markers. However, the presence of various osteogenic biomarkers in stem cells of bone tissue and periosteum of other bone structures of the body has been widely described in the literature [95], which would support the osteogenic capacity of CAPCs and H-MSV obtained from the oral cavity. Despite differences in the expression of certain markers, MSCs from the oral cavity have similar therapeutic potential in bone regeneration. Therefore, the choice of the origin source would seem to depend to a great extent on the availability and ease of obtaining the stem cells. Therefore, oral cavity-derived mesenchymal stromal cells are believed to be a very important and valuable resource for the eventual development of cells for clinical/therapeutic applications in dentistry and medicine due to their easy access and low risk of complications.

### 4.5. Cell Processing

Osteogenic pre-differentiation has been reported to increase the bone repair potency of MSCs [86]. For this reason, several studies seeded stem cells in osteogenic media before implantation [38,40,44]. However, similar results have been obtained by incubating third to fourth-passage stem cells without an osteogenic medium. Cell culture by the enzymatic method has been used for 40 years in the laboratory to isolate cells and is considered the best available method; however, they are not compatible with clinical practice due to the extensive manipulation of the tissue and its long process of preparation [18]. It has been described that their isolation, differentiation, expansion, and proliferation can be avoided, facilitating clinical management [18]. The Rigenera Protocol allows the production of adult mesenchymal stromal cells from a minimum amount of tissue, without the need for cell culture, using long-term enzymatic experimental methods. The Rigenera^®^ device is a technology that performs dental tissue disaggregation and the necessary filtering to obtain an autologous product for immediate application in clinical practice that is capable of promoting bone regeneration [3,13,18,22,25,26,27,31]

#### Biobanking

Biobanks are not-for-profit services that collect, process, store, and distribute biological samples and data. Thanks to their versatility and easy accessibility of the tissue of origin, dental stem cells are a promising resource for both research and clinical applications [96]. The great potential of stem cells for applications in the field of regenerative medicine has been demonstrated, leading to the development of numerous biobanks specialized in their collection [96]. The first tooth bank, named “Three Brackets”, was established at Hiroshima University in 2005. This was followed by the opening of other institutional centers or private companies for storing autologous dental stem cells [97].

Dental stem cell banking has focused on cells contained in the pulp of human deciduous and permanent teeth, especially wisdom teeth. Cryopreservation has proven to be an effective method for the biobanking of tooth and dental pulp [97]. The harvested dental stem cells can be stored as biological insurance for the individual or blood relatives until a relevant disease requires their usage [96].

### 4.6. Complications

Clinical studies have shown a wide clinical potential of MSC application. However, there have been numerous reports of adverse events and side effects associated with MSC therapy [98]. It has been proposed that these reflect aspects of cell processing and culture as they can drastically influence the cell population profile and change protein expression [98]. Furthermore, rare but prominent issues with hemocompatibility have become apparent [99].

However, the clinical studies reviewed here highlight the use of MSCs as being safe and feasible, with only minor side effects. Similar results were obtained in previous reviews [100]. It was shown that the donor sites presented no adverse alterations, with a very similar postoperative period between the groups. On the other hand, for the grafted site, no study reported serious adverse effects or morbidity after stem cell grafting [2,3,13,22,24,25,26,27,28,30,31,33,34,35,36,38,44], apart from the common side effects of regenerative surgeries such as mild-moderate pain and swelling during the first week [2,3,24,27,35,38], and mild hypersensitivity during the following weeks [35]. Postoperative clinical observations revealed healing without functional alterations [2,21].

One study described the development of partial dehiscence in one of the patients [39], and d’Aquino et al. 2009 [2] and Meshram et al. 2018 [40] described complications at the end of the first week. However, these complications gradually abated over time. Only Nagata et al. [37] described a case of progressive alveolar resorption after the sinus lift procedure. Therefore, the biological risk and morbidity of a site grafted with stem cells are minimal. These favorable results may be explained by cell differentiation prior to implantation. Therefore, the use of highly differentiated cells could be essential to avoid adverse effects [90].

Despite the positive results observed in the analyzed studies, MSC therapy remains a risky therapy. Drawbacks of approaches that include the culture of stem cells have prompted investigations into regeneration based on endogenous MSC recruitment with in situ tissue engineering. Stem cell migration is required for morphogenesis and organogenesis during development and for tissue maintenance and injury repair in adults. Successful endogenous MSC recruitment is the first step toward successful tissue regeneration. The identification of stem cell niches in the oral cavity with promising results in bone regeneration lays the foundation for the application of in situ tissue engineering [91].

### 4.7. Bone Repair Evaluation

The healing sequences of the grafted tissues were evaluated by clinical, radiographic, and histological analysis.

#### 4.7.1. Radiographic

A radiographic analysis is the most commonly used test to evaluate success in the bone repair of the grafted site. 2D images such as panoramic [2,36,40,44] and standardized periapical [2,3,22,23,24,26,27,28,30,31,32,33,34,35] radiographs have been used as well as 3D radiographs such as computed tomography (CT) [13,18,31,37,38,39,40].

All periodontal intrabony defects were evaluated by standardized periapical radiographs using parallel techniques and individual custom bite blocks. These images allow the rate of increase in bone height after grafting to be analyzed in two dimensions. Panoramic radiography is used to evaluate large defects such as cystic enucleation [40,44] or alveolar ridges due to the impaction of third molars [2,36]. In the panoramic radiograph, bone regeneration is evaluated by analyzing the change in radiopacity. On the other hand, CT is used to analyze the sections obtained to determine the preoperative defect and postoperative defect through the variation in bone fill volume in large bone defects such as cleft lip and palate [39], cystic enucleation [13,40], and maxillary sinus lift [37].

#### 4.7.2. Clinical Analysis

After surgery, soft tissue healing and normal healing sequences of the grafted tissues were evaluated. For periodontal intrabony defects, various clinical parameters that assess the bone gain were analyzed, such as the tooth mobility before and after grafting [23,24,26,27,28,30,32,34,35]. On the other hand, signs of morbidity, pain, edema, bleeding, inflammation, functionality, and healing of the grafted site were evaluated at different times after bone graft surgery [25].

#### 4.7.3. Histological Analysis

For histological analysis, 2–3 mm trephine biopsy samples were collected from the surgical site [3,25,37,38,39]. Five studies obtained biopsies from the grafted site to later receive an implant [3,25,37,38,39], and two studies obtained the sample for histological evidence without replacement of the bone tissue obtained in the biopsy [22,40]. Histological analysis was performed on different regenerated bone defects such as defects secondary to cystic enucleation [40], alveolar ridges due to third molar impaction [22], atrophic ridges [38], cleft lip, and cleft palate [39]. Histologic results indicated active new bone formation at stem cell-treated sites [25,39,40].

### 4.8. Considerations and Limitations in the Use of Stem Cells

MSCs are cells with the capacity for self-renewal and multilineage differentiation [95]. Oral cavity stem cells have been studied as a possible source of stem cells for bone regeneration. However, there are limitations that must be taken into consideration. Dental stem cells are found in limited amounts, which could make their use in regenerating large defects difficult. Furthermore, dental tissues are specialized tissues that do not undergo continuous remodeling like bone tissue. Therefore, stem cells derived from dental tissues may be restricted in their differentiation potency compared to BMMSCs. Previous studies have demonstrated higher mineral deposition, proliferation rate, and levels of expression of osteogenic marker genes with bone marrow-derived mesenchymal stem cells (BMSCs) compared with oral cavity-derived stem cells, such as DPSCs, BFPSCs, and PDLSCs, in in vitro studies [95,100]. However, it is important to consider that in vivo results have shown that the bone regeneration capacity of oral cavity stem cells is similar to that of BMSCs. Therefore, the pain and morbidity accompanying MSCs obtained from bone marrow are justification for the use of oral cavity stem cells based on successful results reported in the literature. It is important to continue advancing in the study and analysis of the osteogenic capacity of oral cavity stem cells for the development of new therapies to expand therapeutic options.

## 5. Conclusions

Bone regeneration is a complex process that requires the migration of specific cells to form tissues along with available host cells. Stem cells of maxillofacial origin have been proven to be capable of differentiating into different cell types, including bone cells, and therefore have the potential to regenerate damaged tissues and bone structures. However, an additional scaffold complement is required to facilitate the insertion of stem cells into the defect and improve bone regeneration. Seven cell types used for different bone defects have been described: (I) dental pulp stem cells of permanent teeth, (II) stem cells derived from inflamed dental pulp, (III) stem cells from exfoliated deciduous teeth, (IV) periodontal ligament stem cells, (V) cultured autogenous periosteal cells, (VI) buccal fat pad-derived cells, and (VII) autologous bone-derived mesenchymal stem cells. MSCs show differences in the expression of certain markers; however, MSCs from the oral cavity presented similar therapeutic potential in bone regeneration. Therefore, the choice of the source of origin seems to depend to a great extent on the availability and ease of obtaining the required cells.

## Figures and Tables

**Table 1:** The details of the scoping review.

Scoping Review Title	Potential of Oral Cavity Stem Cells for Bone Regeneration: A Scoping Review
Review objective	To analyze and compare the potential of stem cells from different intraoral tissues for use in bone regeneration, focusing on the bone regenerative result achieved with stem cells from the oral cavity.
Review question	What are the stem cells of the oral cavity with osteogenic potential?
Population	This is considered to be the human population, from whom stem cells were extracted from the oral cavity for bone repair.
Concept	The current review studies the trends reported in the literature for oral cavity stem cells and their significance in bone regeneration.
Types of evidence source	Randomized clinical studies, case reports, and case series reported in the literature using stem cells from the oral cavity for bone regeneration.

**Table 2:** Studies using stem cells to repair bone defects.

Study	Type of Study	Stem Cell Niche	Stem Cells	Bone Defect	Scaffold Associated with Stem Cells	Study Groups	Outcomes
**Dental Pulp stem cells (DPSCs) of permanent teeth**							
d’Aquino R. et al., 2009 [2]	Clinical Trial. 7 patients 6 women (w): 1 man (m)	Third molar	DPSCs	Alveolar ridge, secondary to impaction of the third molar on the alveolar cortical plate	Collagen sponge scaffold	Test group (T): DPSCs + Collagen sponge scaffold Control group (C): Sponge without cells	Three months after grafting, one patient’s alveolar bone had an optimal vertical repair and complete restoration of periodontal tissue up to the second molars, as assessed by clinical probing and radiographs. Probing depth analyses revealed an increase in clinical attachment that was quantitatively greater at the T site than at the C site.
Brunelli G. et al., 2013 [13]	Case report. 1 man, 45 years old	Third molar	DPSCs	Sinus lift	Collagen sponge scaffold	(T): DPSCs + Collagen sponge scaffold	The bone density of newly formed bone was approximately twice that of native bone density.
Giuliani A. et al., 2013 [22]	Follow-up of d’Aquino R. et al., 2009 [2], after 3 years. 7 patients 6 w:1 m	Third molar	DPSCs	Alveolar ridge, secondary to impaction of the third molar on the alveolar cortical plate	Collagen sponge scaffold	(T): DPSCs + Collagen sponge scaffold (C): Sponge without cells	Clinical evaluation of bone quality revealed that T was extremely hard compared with the remaining mandible and compared with C. Bone regeneration was always higher at T sites and was responsible for less exposure of second molar roots there than at C sites.
Aimetti M. et al., 2014 [23]	Case report. 1 man, 56 years old	Third molar	DPSCs	Periodontal intrabony defects	Collagen sponge scaffold	(T): DPSCs + Collagen sponge scaffold	The surgical treatment led to clinical improvements at 6 and 12 months compared with baseline. At 6 months, probing pocket depth (PPD) reduction was 5 mm and the clinical attachment level (CAL) gain was 5 mm. After 12 months, these increased to 6 mm and 6 mm, respectively. The 1-year radiograph showed the filling of the intraosseous component of the defect by a bonelike tissue as confirmed during the reentry procedure.
Aimetti M. et al., 2015 [24]	Case series. 4 patients 2 w:2 m Mean age 59.5 ± 8.5 years Range 48–67 years	Third molar	DPSCs	Periodontal intrabony defects	Collagen sponge scaffold	(T): DPSCs + Collagen sponge scaffold	The mean probing depth decreased from 8.0 to 3.3 mm in a year. The mean level of clinical fixation was 11.0 to 6.0 per year. At the end of the observation period, the mean radiographic defect fill was 4.2 ± 1.9 mm.
Monti M., et al., 2016 [25]	6 patients 2 w:4 m Range 22–60 years	Third molar	DPSCs	Third molar post extraction socket	Collagen sponge scaffold	(T): Collagen sponge scaffold + SC (C): Collagen sponge scaffold	Histological analysis showed well-differentiated bone with Haversian system formation at the test site with a very large amount of bone.
Ferrarotti F. et al., 2018 [26]	Randomized clinical trial (ECA). 29 patients 14 w:13 m Range 39–69 years	Teeth	DPSCs	Periodontal intrabony defects	Collagen sponge scaffold	(T): minimally invasive surgical technique (MIST) + DPSDs + collagen sponge (C): MIST + collagen sponge	In the DPSC-treated group, the mean PE reduction and mean CAL gain were 4.9 ± 1.4 mm and 4.5 ± 1.9 mm, respectively, over the 12-month period. The application of DPSC significantly improved the clinical parameters of periodontal regeneration after 1 year of treatment.
Aimetti M. et al., 2018 [27]	Case series. 11 patients 5 w: 6 mMean age 51.2 ± 6.1 years Range 43–59 years	Tooth requiring extraction for impaction or malpositioning as an autologous source for DPSCs	DPSCs	Periodontal intrabony defects	Collagen sponge scaffold	(T): DPSCs + Collagen sponge scaffold	Mean clinical attachment level gain of 4.7 ± 1.5 mm associated with a mean residual probing depth (PD) of 3.2 ± 0.9 mm and remarkable gingival margin stability at 1 year. Complete pocket closure was achieved in 63.6% of the experimental sites. The clinical results were supported by radiographic analysis which showed a bone fill of 3.6 ± 1.9 mm.
Barbier.L et al., 2018 [18]	Double-blind, randomized, split-mouth, controlled clinical trial. 30 patients 22 w: 8 mMean age 23 years Range 18–30 years	Third molar	DPSCs	Impacted lower third molar (ITM) post-extraction sockets	Collagen sponge scaffold	(T): DPSCs+ collagen matrix (C): Collagen matrix	DPSC could not be shown to reduce alveolar bone resorption after mandibular third molar extraction. The response variables were bone density (DB) and bone resorption (SRB). No significant differences were found in the clinical, radiological, and surgical characteristics of the ITM between the T and C groups.
**Stem cells derived from inflamed dental pulp (DPSCs-IP)**							
Li Y. et al., 2016 [28]	Case report. two women patients 30 and 38 years old	Orthodontically extracted, supernumerary, or third molar teeth with irreversible pulpitis	DPSCs-IP	Periodontal intrabony defects	β-tricalcium phosphate	(T): DPSCs-IP + β-tricalcium phosphate.	DPSC-IP could be grafted and had the effect of regenerating new bone to repair periodontal defects 9 months after surgical reconstruction; an effective repairing effect was observed.
**Stem cells from exfoliated deciduous teeth (SHEDS)**							
Hérnandez-Monjaraz B. et al., 2018 [29]	Case report. A 61-year-old man	Dental pulp of a 7-year-old male donor	SHEDS	Periodontal intrabony defects	Scaffold of lyophilized collagen-polyvinylpyrrolidone sponge	(T): SHEDS + scaffold	Densitometry assays revealed an increase in bone mineral density in the walls of the defect at 3- and 6-months post-treatment, which is suggestive of bone tissue regeneration
**Periodontal Ligament Stem Cells (PDLSCs)**							
Feng F., et al., 2010. [30]	Report of 3 cases. 3 men: two 25 and one 42 years old	Third molar	PDLSCs	Periodontal intrabony defects	Bone graft material CALCITITE 4060-2	(T): PDLPs + CALCITITE 4060-2	All patients showed clinical benefits for 72 months after PDLP transplant compared with pre-surgical conditions.
Graziano A. et al., 2013 [31]	Case report. A 32-year-old woman	Dental ligament attached to tooth roots	PDLSCs	Intrabony defects distal to mandibular second molars	Collagen sponge scaffold	(T): Ligament cells periodontal + collagen sponge (C): Collagen sponge	Radiographs taken at 3 and 6 months show significant differences between sites C and T. The latter showed a higher rate of mineralization and complete filling of the coronal component of the defect compared with the control site. The PD before the surgeries was 12 mm for the test and 11 mm for the control; the surgical CAL was 6 mm for the test and 5 mm for the control; after 6 months the PPD was 3 mm for the test and 7 mm for the control.
Vandana K. L. et al., 2016 [32]	Case report. A 27-year-old man	Dental ligament attached to the tooth roots of the third molar	PDLSCs	Periodontal intrabony defects	Gelatin sponge Abgel ^®^©TM	(T): PDLSCs + Abgel ^®^©TM	One-year follow-up revealed 6 mm of gain in the attachment level measured from a fixed reference point (stent) with a negligible change in the gingival marginal position. A change in radiodensity was observed in the area of the defect, suggesting an improvement in the newly formed bone.
Chen F.M. et al., 2016 [33]	ECA. 41 teeth from 30 patients 18–65 years 8 teeth from men: 33 teeth from woman	Third molar	PDLSCs	Periodontal intrabony defects	Bio Oss Graft Materials, guided tissue regeneration (GTR)	(T): PDLSCs+ GTR+ BIO OSS (C): GTR+ BIO OSS	Both groups showed a significant increase in alveolar bone height (decrease in bone defect depth) over time. However, no statistically significant differences were detected between the cell group and the control group.
Shalini H.S. et al., 2018 [34]	ECA. 28 patients 16 w:12 m Mean age 32.635 years	Third molar and alveolar cavity PDLs were removed using a sterile curette	PDLSCs	Periodontal intrabony defects	Abgel ^®^©TM gelatin sponge	(T): open flap debridement (OFD) + A-PDLSc transplant (C) OFD	The result showed a significant reduction in clinical parameters in the T and C groups. The improvement in defect density was statistically significant in the T group. PDLS showed significant gain in clinical fixation level (CAL). Cementoenamel junction (CEJ) at the alveolar bone crest measurement was higher in T.
Sánchez N. et al., 2020 [35]	Quasi-randomized, controlled pilot phase II clinical trial. 20 patients 6 w:14 m 10 test patients (mean age = 48.8, three women] 10 control patients (mean age = 57.5)	Periodontal ligament (PDL) Third molar (11/20) Other molars (3/20) Premolars (3/20) Canines/incisor (3/20)	PDLSCs	Periodontal intrabony defects	Xenogenic bone substitute (Bio-Oss Collagen)	(T): xenogeneic bone substitute (XBS) + PDLSCs (C): XBS + saline).	The most suitable tooth for cell isolation was the third molar (7/10). Cells were successfully differentiated into osteoblasts, chondroblasts, and adipocytes. Application of PDLSC to XBS for treatment of intraosseous lesions resulted in low postoperative morbidity and adequate healing, although no additional benefit was demonstrated compared with XBS alone.
**Cultured Autogenous Periosteal Cells (CAPCs)**							
Schmelzeisen, R. et al., 2003 [36]	Clinical pilot study. 2 patients	Periosteal tissue of the lateral cortex of the mandibular angle.	Periosteal cells	Edentulous atrophic posterior maxillary alveolus	Polymer fleece	(T): periosteal cells + polymer fleece	The results suggest that periosteal-derived osteoblasts in a suitable matrix form lamellar bone within 4 months, allowing reliable implant insertion.
Nagata, M. et al., 2012 [37]	Preliminary clinical study. 25 patients 13 w:12 m Mean age 55.6 years	Periosteum (50 mm ^®^)	cultured autogenous periosteal cell (CAPCs)	Alveolar ridge augmentation and for maxillary sinus lift	Platelet rich plasma (PRP) and autogenous bone of the mandibular region and iliac crest	(T): CAPCs + PRP+ autologous bone (C): conventional bone graft	CAPCs promoted good bone regeneration and reduced the amounts of bone needed for harvest. CAPC resulted in increased alveolar ridges with satisfactory morphology and stable bone volume. CAPC revealed a prominent recruitment of osteoblasts and osteoclasts accompanied by angiogenesis around the regenerated bone. 3D-CT images suggested that bone remodeling was faster in the CAPC bone graft than in a conventional bone graft.
d’Aquino R. et al., 2016 [3]	New protocol 35 patients 21 w:14 m Range 25–64 years	Periosteum (1 to 10 mm)	CAPCs	Alveolar ridge after extraction of a multirooted tooth	Collagen sponge scaffold	(T): collagen sponge + CAPCs (C) collagen sponge	Horizontal resorption at T sites was 38.3% less than at C sites, while vertical resorption at T sites was 36.5% less than at C sites. The combination of micrografts with collagen showed already accelerated processes of ossification in T compared to C at 45 days.
**Buccal Fat Pad-Derived Cells (BFPSCs)**							
Khojasteh A. et al., 2016 [38]	Preliminary study. 8 patients 5 w:3 m Range 25–60 years	Buccal fat pads	BFPSCs	Reconstruction of the atrophic mandibular crest (more than six teeth)	Freeze-dried bone allograft pellets (FDBA)	(T): FDBA + BFP (C) autograft + FDBA + collagen membranes	The mean percentage of newly formed bone was 49.21% in the control group and 65.32% in the test group. The mean bone width gain in the stem cell-treated group was greater than in the control group.
Khojasteh A. et al., 2017 [39]	Randomized prospective clinical trial. 10 patients 3 w:7 m 4 adult patients, 6 were 8 to 14 years	Buccal fat pad-derived mesenchymal stem cells (3 to 5 mL)	BFSCs	Unilateral cleft lip and palate	- Collagen sponge scaffold - Anterior iliac crest (AIC) - Lateral ramus cortical bone plate (LRCP)	Group 1: anterior iliac crest (AIC) bone and a collagen membrane Group 2: lateral ramus cortical bone plate (LRCP) with BFSCs mounted on a natural bovine bone mineral (LRCP + BFSC) Group 3: AIC bone, BFSCs cultured on natural bovine bone mineral, and a collagen membrane (AIC + BFSCs)	Successful healing without fistula or oronasal communication was achieved in all cases. After 6 months, LRCP + BFSC members experienced 69% to 85% new bone formation (BF), while for those in the AIC + BFSC group, it was 70%, 85%, and 90%. In the AIC +BFPSC group, the range of BF was between 75% and 90%, higher than that observed using AIC alone (controls), where it was 65% to 85%. All members of this group were adults (20 to 29 years old) and had a lower regenerative capacity.
Meshram M. et al., 2018 [40]	Pilot study. 5 patients 3 w:2 m Range 18–55 years	Autologous buccal fat pad (5–10 mL of tissue)	BFPSCs	Bone defects secondary to enucleation of cysts or pathological tumors	Drip implantation	(T): BFPSCs	In all patients, thick irregular trabecular bone was discovered during the first month and was replaced by dense compact bone in the third and sixth months. No more bone density increase was observed at 6 months.
**Autologous Bone-derived Mesenchymal Stem Cells (H-MSVs)**							
Pradel W. et al., 2006 [41]	20 patients 5 w:15 m Mean age 45.6 years Range 16–72 years	Mandible or maxilla	H-MSVs	Mandibular cystic bone defects	Demineralized bone matrix Osteovit	(T): autogenous osteoblasts in collagen-based scaffolds (C): autogenous spongiose iliac crest bone was used	After 3 and 6 months there were few differences in bone density between the groups. However, in radiographic controls, ossification was considerably stronger in cysts grafted with tissue-engineered bone after 12 months
Pradel W. et al., 2008 [42]	Report of 6 cases. Mean age 45.2 years Range 38–52 years	Maxilla	H-MSVs	Sinus floor elevation	Demineralised bovine bone matrix (DBBM) and solventdehydrated mineralized bovine bone (SDBB)	(T): (1) DBBM + H-MSVs (2) SDBB + H-MSVs	Histology of the bone cores in the DBBM group at 5 months showed lamellar bone and osteoid, and at 12 months showed fibrous connective tissue. Some resorption of the scaffold was found 5 months after SDBB grafting, and after 12 months cancellous bone formation encapsulating SDBB remnants were observed
Pradel W. et al., 2012 [43]	8 patients 1 w:7 m Mean age 10.3 years, Range 8–16 years	Maxilla	H-MSVs	Unilateral and bilateral cleft lip and cleft palate	Demineralized bone matrix Osteovit	(T): autogenous osteoblasts in collagen-based scaffolds (C): autogenous spongiosa	At 6 months post-surgery, 40.9% of the original cleft defect was ossified in the test group while it was slightly less ossified (36.6%) in the control group.
Redondo L.M. et al., 2017 [44]	Phase I–II trial 11 patients 9 w:2 m Mean age 36 ± 14 years, Range 21–50 years	Cancellous bone (2.8 ± 1.0 mm)	H-MSVs	Maxillary cystic bone defects	A serum cross-linked scaffold (BioMax)	(T): H-MSVs + Biomax (C): contralateral side of cancellous alveolar bone, untreated	Growth tended to be significantly faster in younger patients. Mean increase in bone density of 2.5 times at 7 months after the intervention in the T group; there was no difference in the control group.

**Table 3:** Cell culture methodology and bone regeneration analysis.

Study	Stem Cell Isolation	Post-Surgical Evaluation	Complications
**Dental Pulp stem cells (DPSCs) of permanent teeth**			
d’Aquino R. et al., 2009 [2]	The pulp was mechanically dissociated, then the cells were filtered through a 70 μm filter and cultured in α-minimum essential medium (α-MEM).	Clinical and radiological controls at 7 days, 1, 2 and 3 months - Radiography (RX): panoramic and periapical radiographs - Clinically: edema, inflammation, and functionality. Control once a month until the third month. PD to assess recovered clinical attachment. - Histological observations: 3 months	The patients did not present morbidity or infections after the intervention. One patient suffered from a slight distortion of the mouth opening and an increased level of edema at both sites. All parameters were within normal ranges.
Brunelli G. et al., 2013 [13]	Rigenera ^®^	- Radiographs were taken before and after surgery - RX: Computed tomography (CT) after 4 months	Antibiotic prophylaxis was prescribed, and postoperative medications were indicated. The postoperative course was uneventful.
Giuliani A. et al., 2013 [22]	Stem cells were isolated from the tissue by incubating them with CD34-conjugated microbeads.	Clinical evaluations conducted 3 years after stem cell implantation to assess morbidity, functionality, and bone quality - RX (2D, 3D): 6 months, 1 and 3 years after surgery. Periapical X-ray. - Histological: bone biopsies	Analysis did not reveal the presence of morbidity or infection at the intervention sites. Normal functionality.
Aimetti M. et al., 2014 [23]	Medimax system	Clinical and radiological: a calibrated examiner performed all clinical and radiographic measurements at baseline, and at 6 and 12 months postoperatively. - Clinical: probing pocket depth (PPD), *gingival recession* (REC), clinical attachment level (CAL) - RX: Periapical = radiographic depth of the defect; RA = radiographic angle	Minimal swelling of the soft tissues surrounding the operated areas was observed during the early healing phase.
Aimetti M. et al., 2015 [24]	The pulp tissue was dissociated and passed through disposable filters in a sterile physiological solution to obtain a cell suspension enriched in stem cells.	Clinical and radiographic parameters: before and at 6 and 12 months after the operation by the same calibrated examiner - RX: periapical, standardized - Clinical: plaque index (PI), bleeding on probing (GBI), probing depth (PD), REC, CAL	Minimal swelling of the soft tissues surrounding the operated areas was observed during the early healing phase.
Monti M. et al., 2016 [25]	Rigenera ^®^	First control on day 7 after surgery - Clinical: evaluation of inflammation and functionality - RX: 60 days after grafting X-rays were taken - Histological: a surgical trephine was used to extract a bone sample	Analgesic medication was indicated in the case of postoperative pain. Edema and the presence of inflammation and functionality were evaluated. No complications were observed.
Ferrarotti F. et al., 2018 [26]	Rigenera ^®^	- Clinic: clinical measurements at baseline, 6 and 12 months after surgery. Presence/absence of plaque, morphology of the defect. - RX: at the beginning, 6 and 12 months after surgery. Standardized periapical Rx.	The patients were prescribed analgesic antibiotics. No discomfort or complications were reported.
Aimetti M. et al., 2018 [27]	Rigenera ^®^	Clinical and radiographic parameters were measured at baseline, and at 6 and 12 months after the regenerative therapy by an independent calibrated examiner - RX: standardized periapical Rx - Clinical: Pl, GBI, PD, REC, and CAL	Minimal swelling of soft tissues surrounding the surgical areas was observed during the early healing phase.
Barbier L. et al., 2018 [18]	Rigenera ^®^	To assess bone repair, CT scans were performed 6 months after the third molar extraction	No morbidity was observed during the clinical trial.
**Stem Cells Derived from Inflammatory Dental (DPSCs-IP)**			
Li Y. et al., 2016 [28]	The inflamed pulp samples were digested and cultured in Dulbecco’s modified Eagle media/Nutrient Mixture F-12 (DMEM/F12 1:1).	Clinical evaluation and RX at 1, 3, and 9 months after surgery - Clinical: PI, GBI, PD, REC, CAL, and dental mobility - Rx: periapical	No side effects or uncomfortable feelings appeared in patients after transplantation.
**Stem cells from exfoliated deciduous teeth (SHEDS)**			
Hérnandez-Monjaraz B. et al., 2018 [29]	The dental pulp was dissociated and centrifuged. After centrifugation, the dissociated tissue was resuspended in MEM-α.	Clinical and radiographic evaluation at 3 and 6 months RX: CT Clinical: description of the surgical site, depth of periodontal defect, tooth mobility, bone mineral density	At 3 and 6 months following surgical intervention, the patient showed no signs or symptoms of rejection.
**Periodontal Ligament Stem Cells (PDLSCs)**			
Feng F. et al., 2010. [30]	PDLP cells were cultured in α-MEM supplemented with fetal bovine serum (FBS). CALCITITE 4060-2 bone graft material was added to the surface of the PDLPs.	Clinical and radiographic evaluation: 3, 6, 12, 26, 32, 42, and 72 months - Clinical: PD, REC, attachment gain - RX: Periapical	No patient showed inflammation in the treatment area or any systemic disorder associated with PDLP.
Graziano A. et al., 2013 [31]	Rigenera ^®^	Clinical and radiographic examination - Clinical: at the beginning, 1 week, and 1, 3, 6 months after surgery. Presence or absence of gingivitis, PI, PD, CAL, GMP and GBI - RX: CT before surgery. Standardized periapicals at 3 and 6 months after periodontal surgery	No alterations in the surgical area
Vandana K. L. et al., 2016 [32]	The transplant consisted of soft tissue from the periodontal ligament. No cell culture.	Clinical and radiographic evaluations at baseline, 6 months, and 1 year RX: periapical - Clinical: CAL	Not reported.
Chen F.M. et al., 2016 [33]	The PDLs were digested and cultured in α-MEM.	- RX: periapical. The rate of increase in alveolar bone height was evaluated at 3, 6, and 12 months postoperatively.	None of the patients reported any complication/adverse event other than medium-sized swelling and pain.
Shalini H.S. et al., 2018 [34]	PDL was obtained from the root with a sterile dressing and immediately mixed with a gelatin sponge.	Clinical and RX: at baseline and at 3, 6, 9, and 12 months after the operation by the same calibrated examiner RX: standardized periapical - Clinical: PI, GBI, PD, CAL, GMP and GT	Antibiotic prophylaxis was prescribed, and postoperative medications were indicated. The technique used did not cause any biological risk.
Sánchez N. et al., (2020) [35]	PDL was isolated by root scraping, digested, and cultured.	Clinical and radiographic examinations were recorded at baseline, 6, 9, and 12 months. - Clinical: a trained and calibrated examiner collected clinical data, PPD measurements, CAL - RX: periapical	No serious adverse effects were reported. Mild–moderate pain and swelling during the first week and mild tenderness during the subsequent weeks were the most frequently reported side effects in both groups.
**Cultured Autogenous Periosteal Cells (CAPCs)**			
Schmelzeisen, R. et al., 2003 [36]	The periosteum was digested and cultured in α-MEM	- RX: Panoramic RX at the beginning and at 3 months - Biopsy of the region	Both patients tolerated periosteal harvesting from the mandibular angle. Wounds resulting from tissue replantation and mucosal wounds in the maxilla healed without incident.
Nagata, M. et al., 2012 [37]	Pieces of periosteal sample in culture medium (Medium 199 with Earle’s salts, Invitrogen, Carlsbad, CA)	- RX: CT of the maxillary sinus, before treatment, and at 3 and 12 months after sinus lift - Histological analysis: biopsy at 4 months	No adverse events attributable to the use of CAPC were found. A case with a history of chronic sinusitis showed progressive alveolar resorption after the sinus lift procedure.
d’Aquino R. et al., 2016 [3]	Rigenera ^®^	- Clinical: description of the surgical site - RX: periapical - Histological: surgical trepanning of bone samples	Edema, presence of inflammation, and functionality were evaluated. Healing without alterations after dental extractions. Similar appearances in the alveoli in groups T and C. 7 of 35 subjects required analgesics during the first 2 days.
**Buccal Fat Pad Derived Cells (BFPSCs)**			
Khojasteh A. et al., 2016 [38]	BFP was digested and cultured in α-MEM. Cells from the third to fourth passages were used. Cells were seeded on scaffolds in osteogenic medium.	- Clinical: Soft-tissue healing and the normal healing sequence of the grafted tissue were assessed every 2 weeks. - RX: CT scans were obtained before and five months after implant placement. - Histological: 2 mm trephine biopsy	There was no evidence of inflammation or foreign body reaction.
Khojasteh A. et al., (2017) [39]	The cell suspension of BFP was cultured in α-MEM. MSCs from the third to fourth passage were subjected to the experiments.	- Clinical: soft tissue healing every 2 weeks. - RX: CT–6 months later - 2 mm trephine biopsy for histological analysis	One patient developed partial dehiscence. One case showed partial exposure of the cortical bone of the lateral ramus on the labial side.
Meshram M. et al., 2018 [40]	Cells were differentiated in MSC media for 3 passages and then transdifferentiated into osteoblastic lineage. Cells were cultured in osteogenic media.	Clinical and radiographic: 7 days and 1, 3, 6, 12 months after surgery - RX: panoramic and CT - Clinical: Pain, edema, bleeding, mouth opening, altered sensation and functionality - Biopsy: At the 3rd month bone sampling (biopsy)	Almost all patients had mild pain, edema, and paresthesia at the end of the first week, which gradually decreased. One patient had pain at 1 month, and another patient had paresthesia at 1 month, which steadily improved.
**Autologous Bone-derived Mesenchymal Stem Cells (H-MSVs)**			
Pradel W. et al., 2006 [41]	The explants were suspended in DMEM.	Clinical and radiographic: 3, 6, 12 months after surgery - Rx: panoramic	All the wounds were without any signs of acute infection and healed during follow-up, applying local disinfecting rinses with iodine and saline.
Pradel W. et al., 2008 [42]	The explants were suspended in DMEM.	Clinical and histological	In all patients, primary wound healing was without complications, except for one patient in the SDBB group.
Pradel W. et al., 2012 [43]	The explants were suspended in DMEM.	Radiographic: 6 months after surgery Rx: CT	Wound healing was uneventful in the postoperative period: neither wound dehiscence nor sequestration occurred.
Redondo L.M. et al., 2017 [44]	The explants were suspended in DMEM.	- 3, 4, 6, 8 months after the intervention clinical and radiographic review - Rx: panoramic	In the patients, no signs of inflammation or rejection were recorded in any of the patients.

**Table 4:** Comparative table of oral stem cells and associated scaffolds for bone regeneration.

Cells of the Oral Cavity	Abbreviation	Scaffold Associated with Stem Cells	Repaired Bone Defect
Dental pulp stem cells of permanent teeth	DPSCs	Collagen sponge	Post extraction socket Alveolar ridge, secondary to third molar impaction Sinus lift Periodontal intrabony defects
Stem cells derived from inflamed dental pulp	DPSCs-IP	β-tricalcium phosphate.	Periodontal intrabony defects
Stem cells from exfoliated deciduous teeth	SHEDS	Scaffold of lyophilized collagen–polyvinylpyrrolidone sponge	Periodontal intrabony defects
Periodontal ligament stem cells	PDLSCs	Allograft Xenograft Gelatin sponge Collagen sponge	Periodontal intrabony defects Intrabony defects distal to mandibular second molars.
Cultured autogenous periosteal xells	CAPCs	Collagen sponge Platelet Rich Plasma (PRP) Autograft Polymer fleece	Alveolar ridge augmentation Maxillary sinus lift
Buccal fat pad-derived cells	BFPSCs	Allografts Collagen membrane Autograft	Bone defects secondary to the enucleation of cysts or pathological tumors Reconstruction of the atrophic ridge Unilateral cleft lip and palate
Autologous bone-derived mesenchymal stem cells	H-MSVs	A serum cross-linked scaffold (BioMax) Demineralized bone matrix Mineralized bone	Maxillary or mandibular cystic bone defects Sinus floor elevation Unilateral and bilateral cleft lip and cleft palate

**Table 5:** Oral cavity mesenchymal stromal cell biomarkers.

MSC	Positive Immunoreactivity Biomarkers	Negative Biomarkers	Bone Markers
DPSC	CD10, CD13, CD29, CD44, CD59, CD73, CD90, CD105, CD106, CD117, CD146, STRO-1	CD14, CD19, CD24, CD34, CD45, Human Leukocyte Antigen—DR isotype (HLA-DR)	Dentin sialophosphoprotein (DSPP), dentin matrix protein-1 (DMP-1), osterix (Osx), osteocalcin (OCN), osteopontin (OPN) alkaline phosphatase (ALP), collagen I, Runt-related transcription factor 2 (Runx2)
DPSC-i	CD29, CD44, CD73, CD90, CD105, CD146, CD271,	CD14, CD34, CD45, CD117, HLA-DR	OCN, Type I collagen, OPN, Runx2, bone morphogenetic protein-2 (BMP-2)
SHED	CD13, CD29, CD44, CD73, CD90, CD105, CD106, CD146, CD166, STRO-1	CD11b, CD14, CD18, CD19, CD24, CD31, CD34, CD45, HLA-DR	ALP, DSPP, matrix extracellular phosphoglycoprotein (MEPE), Runx2, OCN, Osx
PDLSC	CD10, CD13, CD26, CD29, CD31, CD44, CD59, CD73, CD90, CD105, CD106, CD140b, CD146, CD166, STRO-1	CD11b, CD14, CD19, CD31, CD34, CD40, CD45, CD79α, CD80, CD86, HLA-DR	ALP, Bone Sialoprotein (BSP), MEPE, OCN
CASp	CD90, CD105, CD73	CD45, CD34	
BFPSCs	CD44, CD90, CD73, CD105	CD45, CD34	Type I Collagen, BMP, OCN
H-MSV	CD73, CD90, CD105, CD166	CD34, CD45, HLA-DR	

Markers listed do not exclude others not listed. CD = Cluster of Differentiation.

## Data Availability

Not applicable.
